# Anatomical Variations in Pulmonary Fissures on Computed Tomography (CT)

**DOI:** 10.7759/cureus.32062

**Published:** 2022-11-30

**Authors:** Nadia Moiz, Saniya Khakwani, Muhammad Asad Ullah, Uzma Azmat, Durr-e- Shahwar, Syed Muhammad Shahnawaz Hyder

**Affiliations:** 1 Family Medicine, Ryds Vårdcentral, Östergötland, SWE; 2 Trust Grade Fellow for Acute Assessment Unit, Whipps Cross University Hospital, London, GBR; 3 Diagnostic Radiology, Memon Medical Institute Hopsital, Karachi, PAK; 4 Diagnostic Radiology, Memon Medical Institute Hospital, Karachi, PAK; 5 Diagnostic Radiology, Indus Hospital, Karachi, PAK

**Keywords:** radiology, anatomical fissure, accesary fissure, ct scan, pulmonary fissure

## Abstract

Objective

To determine the frequency of anatomical variations in lung fissures using computed tomography (CT) at a tertiary care hospital in Karachi, Pakistan.

Methods

A cross-sectional study was conducted in the department of Radiology and Imaging Services at Memon Medical Institute Hospital, Karachi, between November 2021 to April 2022. Patients aged between 15 to 92 years with a completed high-resolution CT scan chest were included. Subjects with no significant structural lung disease that could alter the anatomy were analyzed. Baseline data was gathered using a pre-designed questionnaire, and two qualified radiologists assessed the CT chest images.

Results

A total of 382 subjects participated in this study, out of which 57.1% were males whilst 42.9% were females. The right horizontal fissure was absent in 10 (2.6%) cases. Accessory fissures were seen in 7.33%. The most common fissural variation was azygos fissure (14; 3.7%), followed by superior accessory fissure (six; 1.6%), inferior accessory fissures (four; 1%), and left horizontal fissure (four; 1%). These variations were more common in males. The significant difference was only seen in the superior accessory fissures with respect to gender (P-value<0.05).

Conclusion

This study showed the presence of accessory fissures in 7.33% of patients, the most common being the azygos fissure, irrespective of gender. The absence of normal right horizontal fissures was observed in 2.6% of cases.

## Introduction

Lungs are a pair of vital organs, involved in respiration on either side of mediastinum in the thoracic cavity. It is divided into lobes by a double-layered invagination of the visceral pleura that forms the lobar division of lungs called fissures. Typically, there are two inter-lobar fissures in the right lung and one inter-lobar fissure in the left lung; namely right oblique fissure (ROF), right horizontal fissure (RHF) and left oblique fissure (LOF) [1].

The presence of fissures helps stress-free movement of the lobes and during respiration each lobe and segment expand in a uniform pattern [2]. If the ﬁssures are incomplete, it results in improper lung expansion and hypoxia in patients with intra-thoracic disease. The incomplete or absent lung fissure occurs during embryologic period as a result from either total or subtotal dissolution. On the other hand, if spaces between broncho-pulmonary buds are not eliminated, this results in accessory fissures [3].

The anatomical variations in pulmonary fissures are more frequently diagnosed with advancements in computed tomography (CT) imaging techniques [4]. High-resolution computed tomography (HRCT), post-processing techniques like multi-planar reformation (MPR) and maximum intensity projection (MIP) are also used for frequent investigations [5]. The fissural variations alter the usual patterns of lobar/segmental collapse, resulting in atypical appearances of encysted pleural effusions and unusual spread of airspace infections/neoplasm [6]. Moreover, the knowledge of anatomical variations in the fissures has critical importance prior to surgeries such as lobectomy and segmental resections to avoid undesired complications [7]. The imaging pitfalls which mimic accessory fissures on CT include margins of costal cartilages, fibrotic bands, scars, collapsed visceral pleural margin of a small pneumothorax, margin of bullae, and normal fissure [8].

The normal pulmonary fissures include right oblique fissure, left oblique fissure and right horizontal fissure while the accessory pulmonary fissures include superior accessory fissure, inferior accessory fissure and azygos fissure. A few uncommon fissures include accessory fissures between medial and lateral segments of the right middle lobe, accessory fissures between superior and inferior segments of the lingula on left, accessory fissures between anterior basal and lateral basal segments of the lower lobes on both sides and left horizontal fissure [9].

To date, many studies have shown variations in the fissural anatomy of lungs leading to different lung lobar patterns [1-3,5,9-10]. However, the reviewed literature from this region of the world showed a scarcity of data on anatomical variations in pulmonary fissures. Therefore, the purpose of this study is to determine the frequency of different anatomical variants of lung fissures using CT at a tertiary care hospital in Karachi, Pakistan.

Key messages

The frequency of accessory fissures equates to 7.33%. The most common accessory fissure was azygos fissure followed by superior accessory fissure, inferior accessory fissures and left horizontal fissure. Absent right horizontal fissure was the second most common finding. Fissural variations were more common in males than in females.

Impact of the study

Information regarding fissural variations in our population will have a positive impact among medical professionals for interpretation and modification of lung surgical procedures in clinical cases.

## Materials and methods

This cross-sectional study was conducted in the department of Radiology & Imaging Services, Memon Medical Institute Hospital. Non-probability consecutive sampling technique was used for recruiting the participants. Duration of the study was six months from 1st November 2021 to 31st April 2022. Ethical approval was obtained from the Institutional Review Board (IRB) of Memon Medical Institute Hospital (Ref no. IRB/MMIH/2022/11). Written informed consent was obtained from each study subject and/or their guardian prior to enrolment in the study.

Out of 443 patients who underwent high-resolution CT scan chest for varying respiratory complaints such as shortness of breath, dry or productive cough, fever, coronavirus disease (COVID-19) screening, etc., a total of 382 patients were included. Those with significant airspace/interstitial disease or any other pathology such as lung masses, pleural diseases, mediastinal lesions and chest operations that involved the interlobar fissures, were excluded from the study. The study population included those 15 years of age or older with no significant pulmonary disease that could alter the fissural anatomy. A predesigned study questionnaire was used to obtain baseline details.

All scans were performed using a 16-slice multidetector CT scanner (Alexion, Toshiba) with 350mm collimation, 0.8mm reconstruction interval, 120kVp, and an average 200mA tube current. Following a 30-second delay, scanning was initiated after asking the patient to hold their breath at the end of deep inspiration. Images were acquired from the lung apices up to just below the diaphragm. Two expert radiologists interpreted the HRCT chest images using multi-planar reformats and lung window.

Statistical analysis

Data were analysed using Statistical Packages for Social Sciences (SPSS) version 20 (IBM Corp., Armonk, NY, USA). Descriptive statistics were used to describe the frequency of normal and accessory fissures. Fisher exact test was applied to check the significance between genders. P-value <0.05 was considered to be statistically significant.

## Results

A total of 382 subjects were registered in this study, out of which 218 (57.1%) were males and 164 (42.9%) were females. Frequency of absent right horizontal fissure was observed in 10 (2.6%) cases. Absent right oblique fissure and absent left oblique fissure were not detected in any case (Table 1). 

**Table 1:** Characteristics of studied participants Data presented as n(%)

Parameters		n(%)
Age (years)	15-30	90(23.6%)
	31-45	74(19.4%)
	46-60	76(19.9%)
	61-75	114(29.8%)
	>75	28(7.3%)
Gender	Male	218(57.1%)
	Female	164(42.9%)
Absence of normal fissures	Absent right oblique fissure	0(0%)
	Absent left oblique fissure	0(0%)
	Absent right horizontal fissure	10(2.6%)

**Figure 1:**
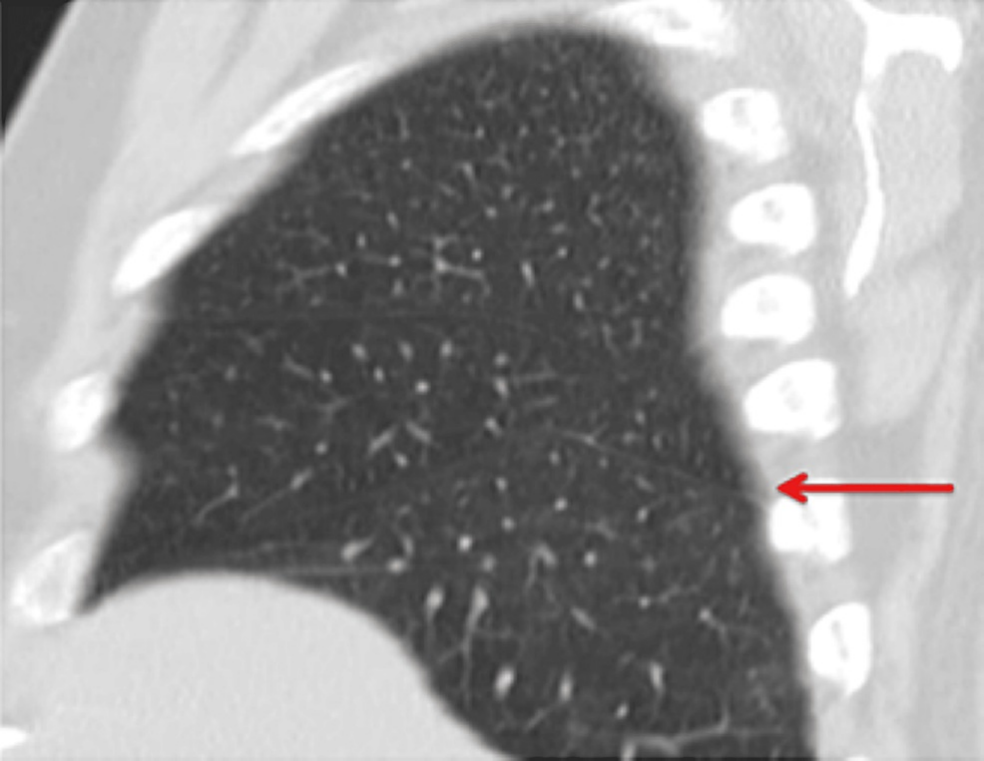
High-resolution computed tomography (HRCT) chest sagittal reformatted image shows thin white line (red arrow) separating the superior segment of the right lower lobe from the remaining segments, the superior accessory fissure

**Figure 2:**
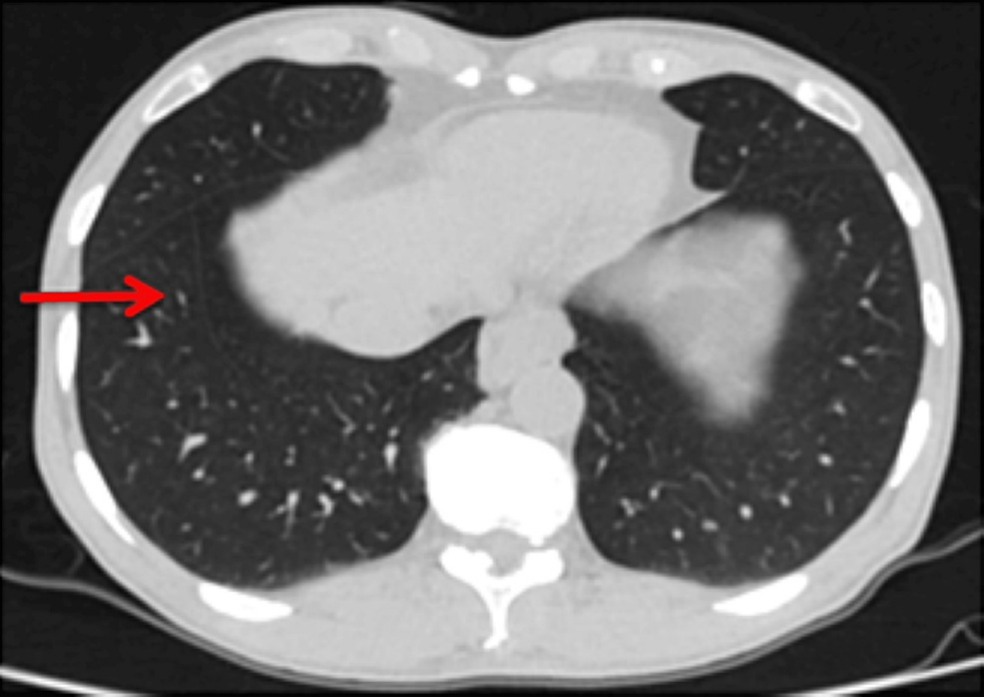
High-resolution computed tomography (HRCT) chest axial image shows slender curvy-linear white line (red arrow) separating the medial basal segment of the right lower lobe from the remaining segments, the inferior accessory fissure

**Figure 3:**
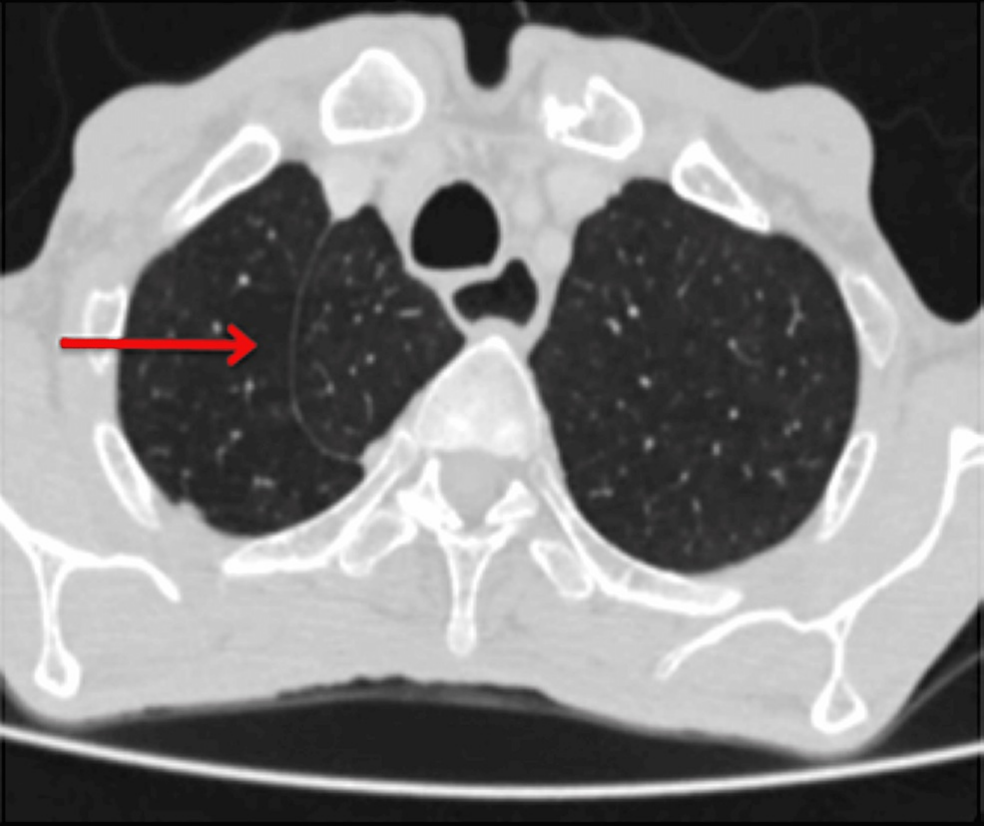
High-resolution computed tomography (HRCT) chest axial image shows curvy-linear white line (red arrow) in the right upper lobe, the azygos fissure.

Superior accessory fissure is the accessory fissure between superior and basal segments of the lower lobe (Figure 1). Inferior accessory fissure separates the medial basal segment from the rest of the segments of lower lobe (Figure 2). The left horizontal fissure is an accessory fissure that separates lingula from the rest of the left upper lobe. The azygos vein penetrates through the upper lobe of the right lung and drags the parietal and visceral pleura with it, thus creating an accessory fissure, known as the “azygos fissure” (Figure 3).

The distribution of different fissural variations is shown in Table 2. A total of 28 (7.33%) fissural variations were seen in this study. The most common fissural variation was azygos fissure (14; 3.7%), followed by superior accessory fissure (six; 1.6%), inferior accessory fissures (four; 1%) and left horizontal fissure (four; 1%).

**Table 2:** Distribution of different accessory fissures Data presented as n(%)

Overall	28 (7.33%)
Azygos fissure	14(3.7%)
Superior Accessory Fissure	6(1.6%)
Inferior accessory fissures	4(1%)
Left horizontal fissure	4(1%)
Accessory fissures between medial and lateral segments of the right middle lobe	0(0%)
Accessory fissures between superior and inferior segments of the lingula	0(0%)
Accessory fissures between anterior basal and lateral basal segments of the right lower lobe	0(0%)
Accessory fissures between anterior basal and lateral basal segments of the left lower lobe	0(0%)

Association of fissural variation with gender is presented in Table 3. Fissural variation was detected in 22 males and six females. Superior accessory fissure and inferior accessory fissure were only present in males. Left horizontal fissure was present in two (0.9%) males and two (1.2%) females. Azygos fissure was observed in 10 (4.6%) males and four (2.4%) females. The significant difference was only seen in superior accessory fissure with respect to gender (P-value<0.05).

**Table 3:** Association of accessory fissures with gender Data presented as n(%); P-value<0.05 consider to be statistically significant

Accessory Fissures	Male	Female	P-value
Overall	22	6	
Azygos fissure	10(4.6%)	4(2.4%)	0.41
Superior Accessory Fissure	6(2.8%)	0(0%)	0.039
Inferior accessory fissures	4(1.8%)	0(0%)	0.138
Left horizontal fissure	2(0.9%)	2(1.2%)	0.999

## Discussion

In this study, we found anatomical variations in the pulmonary fissures in only 7.33% of patients which was lower than Manjunath et al. (22.9%), Ranaweera et al. (68.42%) and other parts of the world (Table 4) [2,9,11-16].

**Table 4:** Comparison of different studies indicating the fissure variation

Study	Variations of fissures
Present study	7.33% fissure variations
Manjunath et al[2]	22.9% fissure variations
Ranaweera et al [16]	68.42% fissure variations
Gebregziabher et al [12]	52.17% right complete oblique fissure 47.82% right incomplete oblique fissure
Mpolokeng et al [9]	5.1% right complete oblique fissure 69.2% right incomplete oblique fissure
Mutua et al [13]	63.16% right complete oblique fissure 36.84% right incomplete oblique fissure
Mote et al [14]	83.4% right complete oblique fissure 16.6% right incomplete oblique fissure

It is a known fact that abnormal embryological development gives rise to variation in lung fissures and lobes [17]. So, if the defect is either complete or incomplete, it results in incomplete or absence of oblique and horizontal fissures [18-19]. In this study, absent right horizontal fissure (2.6%) was commonly observed whilst absent right or left oblique fissure were not noted in any case. Our findings are in line with many researchers who also reported the incidence of absent horizontal fissures to be more common 13.04% and 5% than that of absent right and left oblique fissures 0%, and 2% etc. [20-23]. Jacob and Pillay and George et al. also did not find (0%) absent oblique fissure in anatominal variations [15,24].

The most common fissural variation found in this study was azygos fissure followed by superior accessory fissure, inferior accessory fissures and left horizontal fissure. Yurasakpong et al. in a meta-analysis also found high frequency of presence of azygos fissure [25]. The frequency of superior accessory fissure (1.6%), inferior accessory fissures (1%) and left horizontal fissure (1%) are low in this study in comparison to recent reported study that shows 33.3% and 66.7% superior and inferior accessory fissures [2]. Our results are in contrast to Joshi et al., who found no accessory fissure (0%) in either lung [11]. In accessory fissure a thin white line is detected radiographically that is in close proximity to the major and minor fissure and can only be differentiated by its location. The margins of costal cartilages, fibrotic bands, scars, collapsed lung segments, visceral pleural margin of a small pneumothorax, margin of bullae, and normal fissures may all be misinterpreted as accessory fissures, therefore, these entities must be carefully excluded before actually labeling any linear opacity as an accessory fissure [8]. Taverne et al. also reported that during imaging technique accessory fissures can be overlooked and thus surgical processes become more thought-provoking [26]. Accesary fissures can alter the pattern of lung collapse and deceive the interpreter in accurate prediction of endobronchial lesion. The accessory fissures also control the spread of infection [2]. We also found high incidence of fissural variation in males as compared to females, consistent with previous studies [15]. Manjunath et al. also found 17.5% fissural variations in males and 5.4% fissural variations in females [9]. The reason behind why the fissural variations in males was higher is also debatable.

Environmental and genetic factors may also impact the development of lungs [9]. The accurate knowledge of anatomical variation in lung fissures might help surgeons as well as radiologists in making diagnoses correctly, modifying surgical procedures and prevent postoperative hemorrhage and other complications.

Limitations and future recommendations

Our observation of low fissural variation may be due to the small sample size, which is the limitation of this study. Future cross-sectional studies are still required on larger sample sizes.

## Conclusions

Overall the frequency of accessary fissure was found to be low in the Pakistani population. The most common were the azygos fissure and absent right horizontal fissure, irrespective of gender. Knowledge regarding variations in the pulmonary fissures and lobes is important among medical professionals for interpretation and modification of a surgical procedure in clinical cases concerning the pathology of lungs.
